# Flexible frequency selective metamaterials for microwave applications

**DOI:** 10.1038/srep45108

**Published:** 2017-03-21

**Authors:** Bo Gao, Matthew M. F Yuen, Terry Tao Ye

**Affiliations:** 1 Department of Mechanical Engineering, Hong Kong University of Science and Technology, Clear Water Bay, Kowloon, Hong Kong; 2 SYSU-CMU Joint Institute of Engineering, SYSU-CMU International Joint Research Institute, Guangdong, China

## Abstract

Metamaterials have attracted more and more research attentions recently. Metamaterials for electromagnetic applications consist of sub-wavelength structures designed to exhibit particular responses to an incident EM (electromagnetic) wave. Traditional EM (electromagnetic) metamaterial is constructed from thick and rigid structures, with the form-factor suitable for applications only in higher frequencies (above GHz) in microwave band. In this paper, we developed a thin and flexible metamaterial structure with small-scale unit cell that gives EM metamaterials far greater flexibility in numerous applications. By incorporating ferrite materials, the thickness and size of the unit cell of metamaterials have been effectively scaled down. The design, mechanism and development of flexible ferrite loaded metamaterials for microwave applications is described, with simulation as well as measurements. Experiments show that the ferrite film with permeability of 10 could reduce the resonant frequency. The thickness of the final metamaterials is only 0.3mm. This type of ferrite loaded metamaterials offers opportunities for various sub-GHz microwave applications, such as cloaks, absorbers, and frequency selective surfaces.

The term metamaterials covers a broad category of materials including both resonant and non-resonant artificial structures consisting of specially arranged sub-wavelength unit cells. The concept of metamaterials was first introduced by Soivet physicist Veselago in 1968^1^. Veselago discovered that specially arranged structures have a number of unique properties like negative permittivity and permeability, as opposed to conventional material that exist in nature. In 1996, Pendry proposed metal mesh which demonstrates an ultra-low plasma frequency^2^. This discovery opens the new research area of metamaterials. This type of material is widely studied for cloaks, EM superlens, surface antenna, as well as absorbers^5^,^6^,^7^,^8^,^9^,^10^,^11^,^12^,^13^,^14^,^15^,^16^,^17^,^18^,^19^,^20^,^21^,^22^.For the past two decades, the metamaterial developments are mostly for optics and EM applications in high frequency range (>10 GHz) with thick and rigid cell structures. Little research is conducted for sub-GHz frequency and flexible metamaterial designs.

To lower the resonant frequency with flexible metamaterial structures, more and more efforts are put on designing the complex structures. The sub-GHz mushroom like metamaterial was studied in early 2000s^3^,^4^. This is a three layer mushroom-like structure. The top and bottom layer is made of conductive metal. These two layers are connected to the ground plane by conducting vias The inner layer adds the intrinsic capacitance to lower down the resonant frequency. The advantages of this novel material, including low profile, light weight and low fabrication cost had drawn significant attentions during the past ten years.

In addition to the mushroom-like metamaterials, a new type of low frequency metamaterials, called fractal metamaterials, has also been explored, such as space filling curve or fractal single layer structures. Because the fractal structure could use the space efficiently, and the metal traces could be fully placed within a unit cell, the intrinsic capacitance and inductance of unit cell is higher than other metamaterials design. All these unique properties of fractal metamaterial have attracted tremendous amount of research interests in recent years.

Another widely studied metamaterial structure is SRR (Split Ring Resonator) which is the design originally proposed for strong artificial magnetism. Each SRR is composed of two concentric split rings with the openings at the opposite directions, as illustrated in Fig. 1. The equivalent circuits of an SRR can be regarded as an LC circuit with the natural resonant frequency. Within a certain frequency range, the magnetic flux flowing through an SRR induces a strong circulating current, resulting in an effective magnetic moment. This induced magnetic moment responds in phase or out of phase with respect to the external magnetic field.

In our approach, SRR was adopted for low resonant frequency and flexible structure design due to the cells’ strong magnetic flux and low profile. To further decrease the resonant frequency, ferrite films with higher permeability and permittivity had been applied to the SRR backplane to increase the cell’s intrinsic inductance. The simulation as well as the prototype has demonstrated that SRR based metamaterial structure effective lower the resonant frequency to sub-GHz range, in the meantime, the low profile geometry also makes it possible to be fabricated on a flexible substrate with ferrite films used as backplane.

Ferrite materials used in metamaterial structures had been proposed by earlier researchers, however, instead of ferrite films, ferrite rod structures were used, with permeability in the order of thousands. Furthermore, the ferrite core used in the rod is big and difficult to fabricate, which makes it infeasible for flexible and commercial applications. This is the first paper discussing the usage of ferrite film to achieve flexible metamaterials.

## Results

The traditional SRR (split ring resonator) is an artificially prepared structure which exhibits strong magnetic coupling to an electromagnetic field. The unit cell of SRR is consisted of an enclosed loops made by metal with splits at ends. The SRR is usually fabricated on printed circuit board (PCB) with 2D planar structure. However, the size of traditional SRR is relatively large for sub-GHz application. The standard PCB is also thick and rigid. To reduce the unit cell size and build flexible SRR metamaterials, the ferrite film is attached on polyimide based flexible printed circuit (FPC) to lower down the resonant frequency. The ferrite film is made of ferrite particles mixed in polymer matrix with permeability of 10 and permittivity of 10. The ferrite film can both increase the SRR’s capacitance and inductance. The high capacitance can lower down the resonant frequency into the sub-GHz range. Figure 2 shows the structure of proposed SRR. To demonstrate the enhancement of ferrite film, we fabricated two samples for comparison, i.e., SRR without ferrite film used as a control samples, and SRR structure with ferrite film.

**Figure 1:**
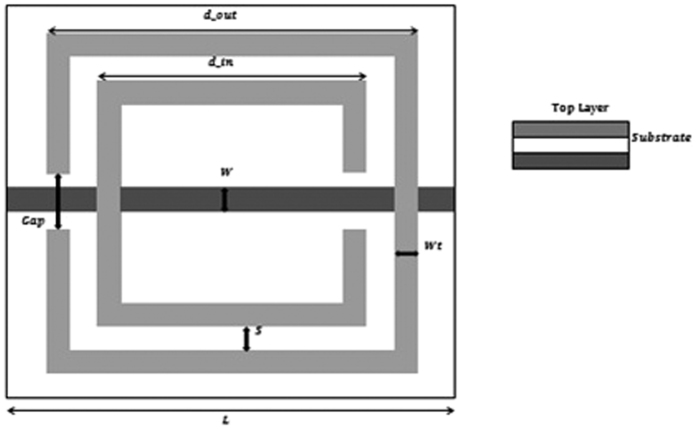
Schematics of split ring resonator (SRR). The SRR is prepared on a single layer of standard printed circuit board (PCB).

In this paper, the designed dimensions of the SRR are given by as shown in Fig. 1:

- D_out: outer length of SRR
- D_in, inner length of SRR
- Gap: split width in rings
- Wt: width of ring trace
- S: distance between rings
- L: unit cell size
- t: thickness of substrate
- w: thickness of metal rod
- ε: The permittivity of substrate

The D_out, D_in, Gap, Wt, S, L, t, w, and ε are 22mm, 15mm, 3mm, 2mm, 1.5mm, 25mm, 0.1mm, 1.4mm, and 3.5 respectively.

### Simulation

**Figure 2:**
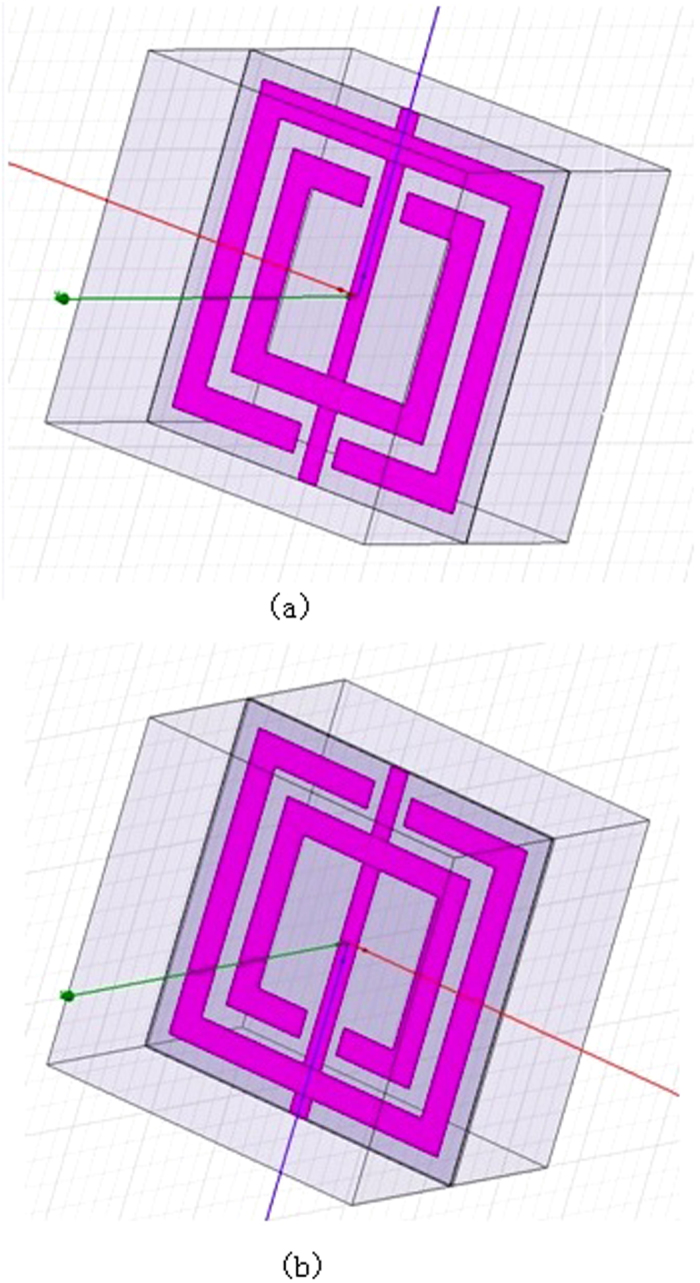
Simulation setup of (**a**) unit cell of SRR. Two wave port excitation is placed on the left and right side to excite the SRR unit. (**b**) unit cell of SRR-Ferrite. A layer of ferrite film of 0.3mm is placed on the top of SRR surface. The ferrite film with a permittivity of 10 and permeability of 10 could increase the intrinsic inductance of SRR unit cell.

The metamaterial is analyzed in finite element method software, Ansys HFSS. The unit cell of this metamaterial designed is simulated with periodic boundary conditions in finite element model. The transmission and reflection of this unit cell is simulated to acquire Scanter parameter. The SRR is fabricated on the PCB board, where the dielectric material is FR4 with dielectric constant of 4.4. The design layout of SRR is shown in Fig. 2. The unit cell size is 25mm. As mentioned above, the SRR is defined as a periodic and infinite plate. Periodic boundary conditions are used to simulate infinite plate. In this case, the perfect electric (E) boundary and perfect magnetic (H) boundary are used as periodic boundary, as shown in figure below. A perfect E boundary is used to represent a perfectly conducting surface in the structure. The electric field is assumed to be normal to these surfaces. A perfect H boundary represents a surface on which the tangential component of the H-field is the same on both sides. For surfaces on the outer surface of the model, this condition results in a boundary that simulates a perfect magnetic conductor in which the tangential component of the H-field is zero. Two wave ports are also assigned to excite this SRR structure. This wave port is defined as a waveguide with the same cross section of port. Wave ports are placed on the left and right interface to provide a window that couples the model device to the external.

**Figure 3:**
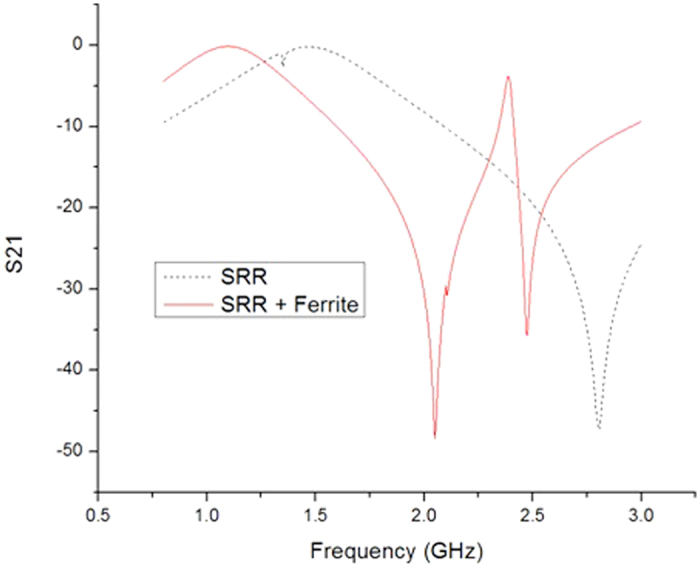
Simulation results of S21 parameter of SRR unit cell and SRR-ferrite unit cell. The resonant frequency of SRR unit cell is at around 2.8 GHz. For the SRR unit cell loaded by ferrite, the resonant frequency is at around 2.05 GHz.

This simulation is analyzed at 2 GHz and the simulation results are shown in Fig. 3. The resonant frequency is defined as the frequency of minimum S21. It is can be seen that the resonant frequency of this SRR is at around 2.8 GHz where the S21 is at its minimum. The resonant frequency of Ferrite-SRR is at 2.05 GHz as shown in Fig. 3. It is clearly found that the resonant frequency can be decreased by around 27% by add a layer of 0.3 mm thick ferrite film of 10 permeability and 10 permittivity. The resonant frequency of metamaterial could be significantly reduced by adding a layer of ferrite film.

### Prototype and measurements

**Figure 4:**
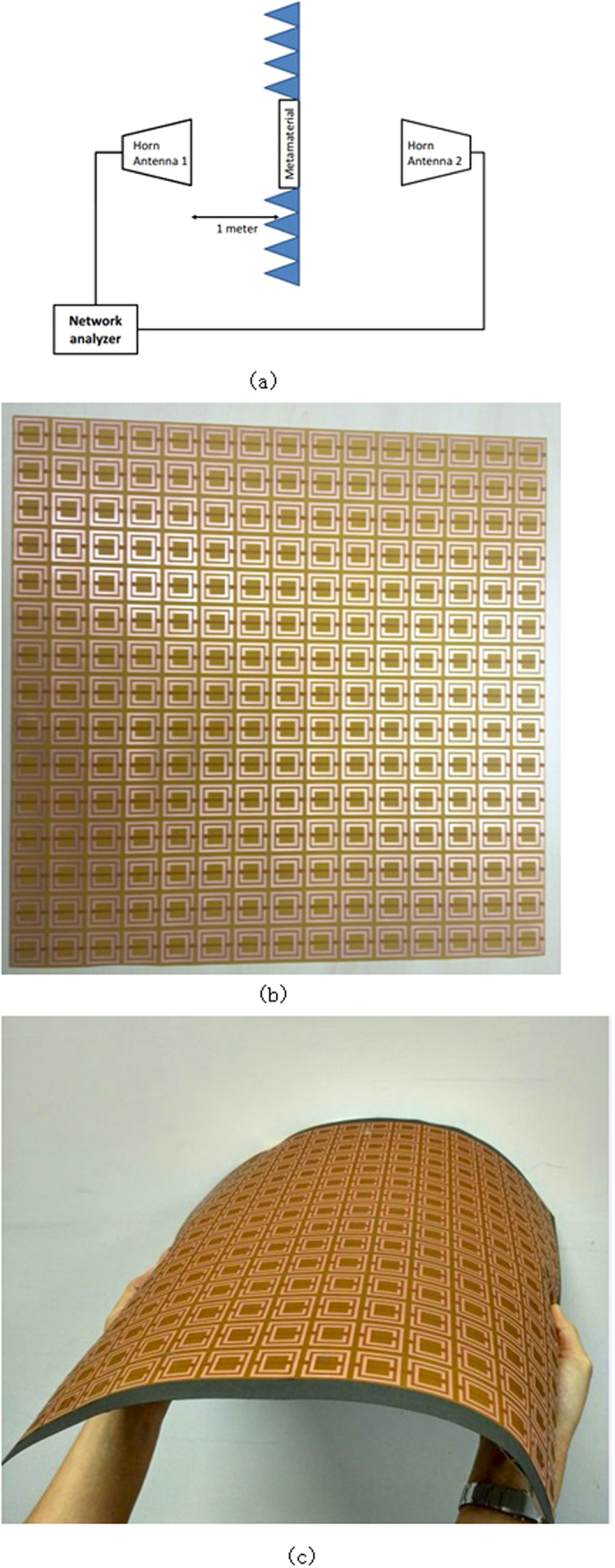
Measurement setup (**a**) schematic of the measurement setup. The metamaterial is surrounded by microwave absorbers. Two horn antennas operating from 0.8GHz-8.0 GHz are used to transmit and receive signals. The network analyzer connects the two horn antennas. The distance between metamaterial and horn antenna is 1 meter. (**b**) The photo of test setup. The measurement is conducted in a anechoic chamber to eliminate external measurement noise. Left one is the SRR slab and right is the SRR covered by 0.3mm thick ferrite film.

As discussed above, the metamaterial property is retrieved from the S parameter of unit cell. In the measurement setup, we use horn antennas to conduct the transmission testing to get S21 data, as well as the transmission data. The setup of measurement is as shown in Fig. 4. Two horn antennas operateing from 0.8 GHz to 8 GHz are used. One is used as a transmitter and the other is used as a receiver. The antenna is set 1 meter away from the metamaterial to make sure the incident wave is plane wave. To eliminate external noise, this whole setup is situated inside an anechoic chamber and the material under test is covered by microwave absorbers. The testing structure is supported by foam stand as shown in Fig. 4.

The system needs to be calibrated prior to S-parameter measurement in order to remove the systematic error. The transmission parameter measurement needs to be calibrated as well in order to eliminate the influence of transmission path. For S21 calibration, the absence of metamaterials is used as the baseline transmission calibration. The measurement setup is shown in Fig. 4.

**Figure 5:**
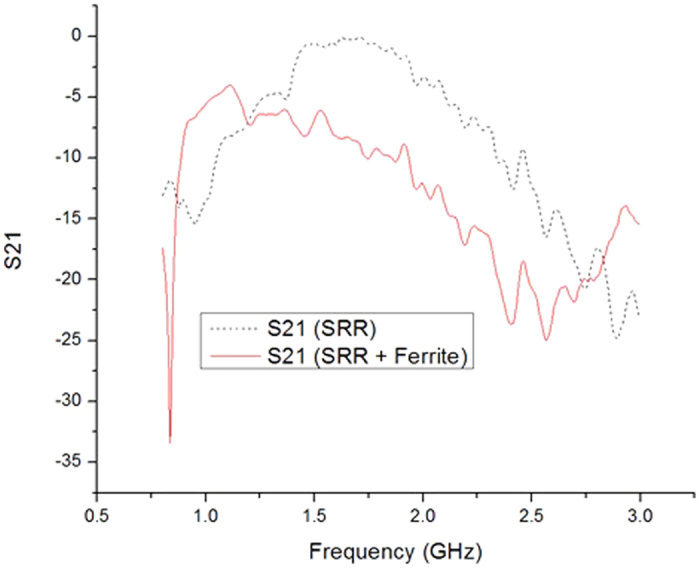
Measurement results of SRR slab and SRR slab covered by a 0.3 mm thick ferrite film. The resonant frequency of SRR slab only is at around 2.9 GHz. The resonant frequency of ferrite covered SRR is at 2.56 GHz.

The measure results are shown in Fig. 5. The resonant frequency of SRR slab is at 2.9 GHz and the resonant frequency of SRR-ferrite is at 2.56 GHz. The measurement results also demonstrated that the ferrite film can decrease the resonant frequency by around 12%.

## Discussion

We also noticed there is a different of scattering parameters between simulation and measurement results. The comparison between simulation and measurement is listed in Table 1. This difference might be introduced by a couple of factors. First, the material used in simulation is not accurate. In the simulation, the material property is from HFSS’s library and materials specifications. The prototype’s property, especially the ferrite film, might be different from the datasheet and library. Second, the conducting material used in the simulation is 2D planar copper. The material thickness is not considered, which might also introduce errors. Last, the fabrication of samples might also induce fabrication errors. In addition, the ferrite film itself could also introduces loss. In Fig. 5, the maximum S21 of SRR only is around 0 dB. For the SRR + Ferrite film, the maximum S21 is around - 4 dB. We can have a rough idea that the ferrite film introduces a 4 dB loss. The minimum S21 of “SRR + ferrite” is around −25dB. That means the loss introduced by ferrite is small compared to the SRR’s frequency selective properties.

In this paper, we demonstrate a ferrite film loaded metamaterials for microwave applications. The ferrite film used is widely used for EMC/EMI and other application. The ferrite film is a low cost and easy to fabricate solution for metamaterials. By applying the ferrite film, the resonant frequency can be reduced by 12%. Since this ferrite film is flexible, it will also be easy to realize low cost, low frequency metamaterials. It is believed that this kind of ferrite loaded metamaterials could find many application in sub-GHz microwave area, like frequency tuning, shielding, and radiation enhancement.

## Methods

The split ring resonators are two rings in the unit cell. The equivalent circuit model of the SRR is as shown in figure The impedance of the surface for a perpendicular incident electromagnetic wave as given in is


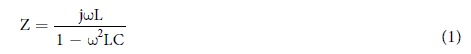


Where Z is the impedance of surface, ω is the frequency of incident electromagnetic wave, L is the sheet inductance and C is the sheet capacitance. So the surface impedance becomes infinite when the frequency is as below:


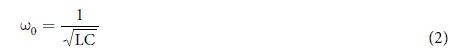


In order to lower the resonant frequency, either the equivalent capacitance or the equivalent inductance of the unit cell needs to be reduced.

## Additional Information

**How to cite this article:** Gao, B. *et al*. Flexible frequency selective metamaterials for microwave applications. *Sci. Rep.* **7**, 45108; doi: 10.1038/srep45108 (2017).

**Publisher's note:** Springer Nature remains neutral with regard to jurisdictional claims in published maps and institutional affiliations.

## Figures and Tables

**Table 1:** Comparison of scattering parameters.

Name	Resonant Frequency (Simulation)	Resonant Frequency (Measurement)
SRR	2.8 GHz	2.9 GHz
SRR-ferrite	2.05 GHz	2.56 GHz

The resonant frequencies of SRR and SRR-ferrite are listed. The measured resonant frequency is lower than the simulation results. The difference between simulation and measurement for SRR and SRR-ferrite is 3.6% and 25%.
